# Antennal RNA-sequencing analysis reveals evolutionary aspects of chemosensory proteins in the carpenter ant, *Camponotus japonicus*

**DOI:** 10.1038/srep13541

**Published:** 2015-08-27

**Authors:** Masaru K. Hojo, Kenichi Ishii, Midori Sakura, Katsushi Yamaguchi, Shuji Shigenobu, Mamiko Ozaki

**Affiliations:** 1 Department of Biology, Graduate School of Science, Kobe University, Kobe, Japan; 2 Department of Biological Sciences, Graduate School of Science, The University of Tokyo, Tokyo, Japan; 3 NIBB Core Research Facilities, National Institute for Basic Biology, National Institutes of Natural Sciences, Okazaki, Japan; 4 Department of Basic Biology, School of Life Science, Graduate University for Advanced Studies, Okazaki, Japan

## Abstract

Chemical communication is essential for the coordination of complex organisation in ant societies. Recent comparative genomic approaches have revealed that chemosensory genes are diversified in ant lineages, and suggest that this diversification is crucial for social organisation. However, how such diversified genes shape the peripheral chemosensory systems remains unknown. In this study, we annotated and analysed the gene expression profiles of chemosensory proteins (CSPs), which transport lipophilic compounds toward chemosensory receptors in the carpenter ant, *Camponotus japonicus*. Transcriptome analysis revealed 12 CSP genes and phylogenetic analysis showed that 3 of these are lineage-specifically expanded in the clade of ants. RNA sequencing and real-time quantitative polymerase chain reaction revealed that, among the ant specific CSP genes, two of them (*CjapCSP12* and *CjapCSP13*) were specifically expressed in the chemosensory organs and differentially expressed amongst ant castes. Furthermore, *CjapCSP12* and *CjapCSP13* had a ratio of divergence at non-synonymous and synonymous sites (dN/dS) greater than 1, and they were co-expressed with *CjapCSP1*, which is known to bind cuticular hydrocarbons. Our results suggested that *CjapCSP12* and *CjapCSP13* were functionally differentiated for ant-specific chemosensory events, and that *CjapCSP1*, *CjapCSP12*, and *CjapCSP13* work cooperatively in the antennal chemosensilla of worker ants.

Social insects develop sophisticated societies in which local interactions among individuals facilitate the coordination of group-level activity. Communication among individuals is essential in these social organisations, and thus analysis of its molecular basis helps us understand the evolution of social organisation. Ants are an ecologically successful group of species, and they exhibit a variety of social organisations and behaviours^1^. They communicate principally by chemical signals^2^, while several neuroanatomical features give them an elaborate chemosensory processing ability^3^,^4^,^5^. Furthermore, recent comparative genomic approaches^6^,^7^,^8^ have revealed that chemosensory genes are diversified in ant lineages. The number of chemosensory receptor gene families is relatively high in ants, i.e., more than 400 chemoreceptor genes (*ORs*, *GRs*, and *IRs*) are present in the ant genomes^9^, compared to 183 in the European honeybee, *Apis mellifera*^10^, genome. These results suggest that the diversification of chemosensory genes is crucial for the complex social organisation and ecological dominance of ants^6^,^9^. However, how such diversified genes shape the peripheral chemosensory systems remains unknown.

Insect antennae are the principal chemosensory organs that have various chemosensilla on the flagellum. Chemosensilla are also found on various appendages, such as the maxillary and labial palps, as well as on the legs^11^. Generally, an aqueous lymphatic fluid surrounds the receptive membranes of peripheral receptor neurons in insect chemosensilla. Thus, water-insoluble lipophilic compounds require water-soluble carriers to access those receptive membranes^12^. Odorant binding proteins (OBPs) and chemosensory proteins (CSPs) are responsible for the solubilisation and transportation of lipophilic compounds through the aqueous sensillar lymph^13^,^14^. The odorants carried by OBPs and CSPs are decoded when olfactory receptors (ORs) and possibly, gustatory receptors (GRs) selectively bind adequate stimulus chemicals. Binding proteins such as OBPs and CSPs are small, globular, water-soluble proteins with an inner hydrophobic pocket for lipophilic ligand binding, characterized by a specific domain of six α-helices. They are transcribed in and secreted from the supportive cells that surround the receptor neurons at the base of chemosensilla^12^.

CSPs are major antennal proteins in ants^15^,^16^,^17^. In *Camponotus japonicus* (Hymenoptera: Formicidae), *CSP1* is highly expressed in the antennae and carries cuticular hydrocarbons^16^, which convey various information signals related to social organisation^18^,^19^,^20^,^21^,^22^. Recent studies have indicated that specific CSP gene clades are diversified in ants^8^,^23^,^24^. Annotation of CSP genes from the genomes of 8 different ant species revealed that the CSP gene family consists of two major clades^8^,^24^. One clade is a conserved group shared among different insect orders. These genes consist of 8 orthologous groups (*CSP1–8*), and they have evolved through purifying selection. The other clade has repeatedly expanded in individual ant lineages, and the expanded copies have probably descended from *CSP1*^8^,^24^. This suggests that ant-specific CSPs have distinct functions related to ant chemical communication^8^,^23^, and the analysis of comprehensive CSP distribution among the chemosensory organs may provide a clue to understanding their physiological roles^24^,^25^.

In this study, we conducted RNA sequencing (RNA-seq) and real-time quantitative polymerase chain reaction (qRT-PCR) to characterize and determine the caste- and tissue-specific expression of CSP genes in carpenter ant, *C. japonicus*. We subsequently determined the spatial localisation of antennal CSP transcripts in worker ants. In the light of our findings, we discuss how ant-specific CSPs shape the peripheral chemosensory systems in ants.

## Results

### Transcriptome sequencing and assembly

A comprehensive catalogue of genes expressed in *C. japonicus* antennae was built using RNA-seq. We constructed antenna-specific and whole-body RNA-seq libraries using mRNA isolated from three ant castes, males, alate queens, and workers. Sequencing using Illumina HiSeq2000 platform yielded 358.9 million 101-bp paired-end sequence reads. Cleaned reads from these libraries were assembled together using Trinity^26^, an RNA-seq *de-novo* assembler, resulting in 156,446 contigs that grouped into 68,319 isoform clusters (i.e., unigenes). From the transcriptome sequences, we extracted and selected 36,496 non-redundant open reading frames (ORFs), ranging from 150 bp to 30 kbp with N50 of 1,524 bp. The ORF set was used as a reference coding-sequence catalogue of *C. japonicus* for downstream analysis.

### CSP genes

**Figure 1:**
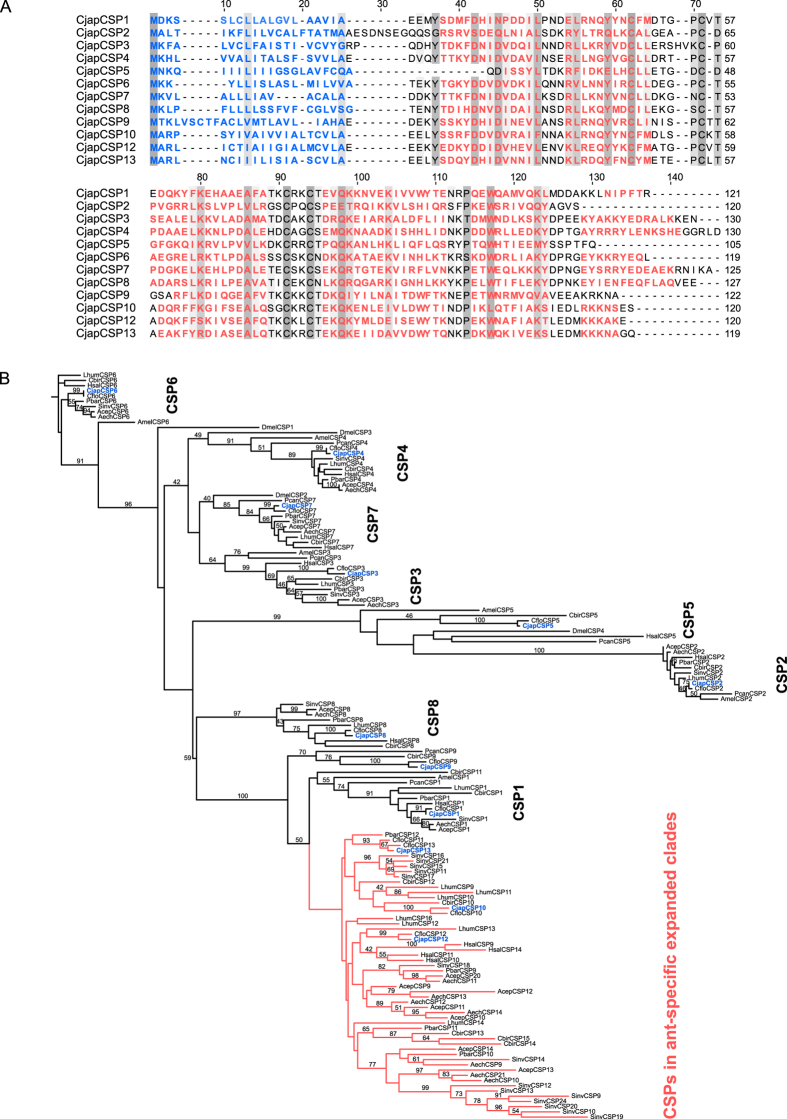
Twelve chemosensory proteins (CSPs) identified from the antennal transcriptome of *Camponotus japonicas*. (**A**) Multiple alignments of *C. japonicus* CSP (CjapCSP) sequences. Conserved amino-acid residues are highlighted based on the degree of conservation. Blue letters indicate signal peptide sequences predicted by SignalP program, and red letters indicate helical structure predicted by structural modelling using MbraCSPA6 as reference. (**B**) Maximal likelihood tree of protein sequences from 9 ant species, *Drosophila melanogaster*, *Polistes canadensis*, and *Apis mellifera*. Bootstrap values greater than 40 are indicated. Red lines indicate expanded ant-specific CSP genes.

tBLASTx search using CSP sequences of other insects allowed us to identify 11 putative CSP genes in the reference coding-sequence catalogue of *C. japonicus*. In addition, a hidden Markov model (HMM)-based motif search for OS-D superfamily (pfam03392) detected another CSP gene candidate. All these CSP sequences were manually inspected and corrected for any computer prediction errors (i.e., wrong start codon before the signal peptide sequences). We also evaluated the *CjapCSP* nucleotide sequences using cDNA cloning followed by Sanger sequencing. Finally, we identified 12 CSP genes from the *C. japonicus* transcriptome, with a predicted length of 84 to 111 amino acids without signal peptide sequences (Fig. 1A, Supplemental Dataset S1 & S2). The 12 CSP genes were designated as *CjapCSP1*–*10*, *CjapCSP12*, and *CjapCSP13*, in accordance to CSP nomenclature for ants proposed by McKenzie *et al*.^24^ and the CSP numbering described by Kulumni *et al*.^8^,^23^.

Multiple alignments revealed that two disulphide bonds were conserved among the 12 CjapCSPs. Protein modelling revealed that all CSPs had a six-helical structure, except for CjapCSP2, CjapCSP5, and CjapCSP9, which had a five-helical structure (Fig. 1A). Phylogenetic analysis showed that the ant CSP family was constructed with ancestral CSPs shared with various other insect species and consisted of 8 orthologous groups (Fig. 1B). Ant-specific duplications occurred in the *CSP1* lineages, leading to species-specific CSP gene groups (Fig. 1B). Although *CfloCSP9* is considered an expanded group of ant-specific genes^8^, in our phylogeny and that of McKenzie *et al*.^24^, the CSP9 group was clustered between the ancestral clade and the CSP1 clade along with orthologs from *Cerapachys biroi* and *Polistes canadensis*. We also found that *CjapCSP10*, *12*, and *13* were included in expanded ant-specific genes. Additionally, all *CjapCSP*s showed one-to-one orthology with those of *Camponotus floridanus*, except for *CfloCSP11* (Fig. 1B). To detect the genes under positive selection, we estimated the selective pressure on the 12 CSP genes of *C. floridanus* and *C. japonicus*. In the branch-site test of positive selection, the branch of the expanded ant-specific CSP genes was significant (Table 1; LRT, q = 0.037), although all the pairwise branches of *Camponotus* CSP genes, including *CSP10*, *12*, and *13*, were non-significant (Table 1; LRT, q > 0.05). The maximum likelihood estimation of non-synonymous and synonymous sites (dN/dS) ratio of *Camponotus CSP1–8* and *CSP10* was lower than 1, while that of *Camponotus CSP9, 12* and *13* was greater than 1 (Table 2).

### Differential expression of CSP genes among castes

**Figure 2:**
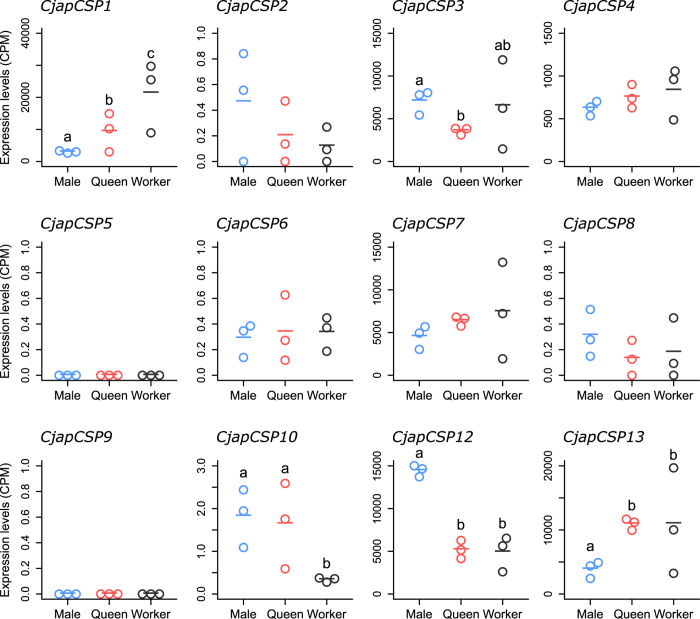
Expression of each chemosensory protein (CSP) gene in the antennae of *Camponotus japonicus* males, alate queens, and workers. Expression levels are indicated as normalised counts per million (CPM) calculated from RNA-sequencing analysis. Significant differences are indicated by different letters (False Discovery Rate (FDR) < 0.05). All analyses were performed in triplicate.

Based on RNA-seq, we compared the expression profiles of CSP genes in the antennae of *C. japonicus* males, alate queens, and workers (Fig. 2). *CjapCSP1*, *3*, *10*, *12*, and *13* showed quantitatively different expression among castes (FDR < 0.05). *CjapCSP1* and *13* were highly expressed in female castes (alate queens and workers), while *CjapCSP12* was highly expressed in males. *CjapCSP*3 was less expressed in alate queens compared to males. *CjapCSP10* was highly expressed in the reproductive castes (alate queens and males), although the expression level was relatively lower than that of other differentially expressed CSP genes.

### Differential expression of CSP genes among workers

**Figure 3:**
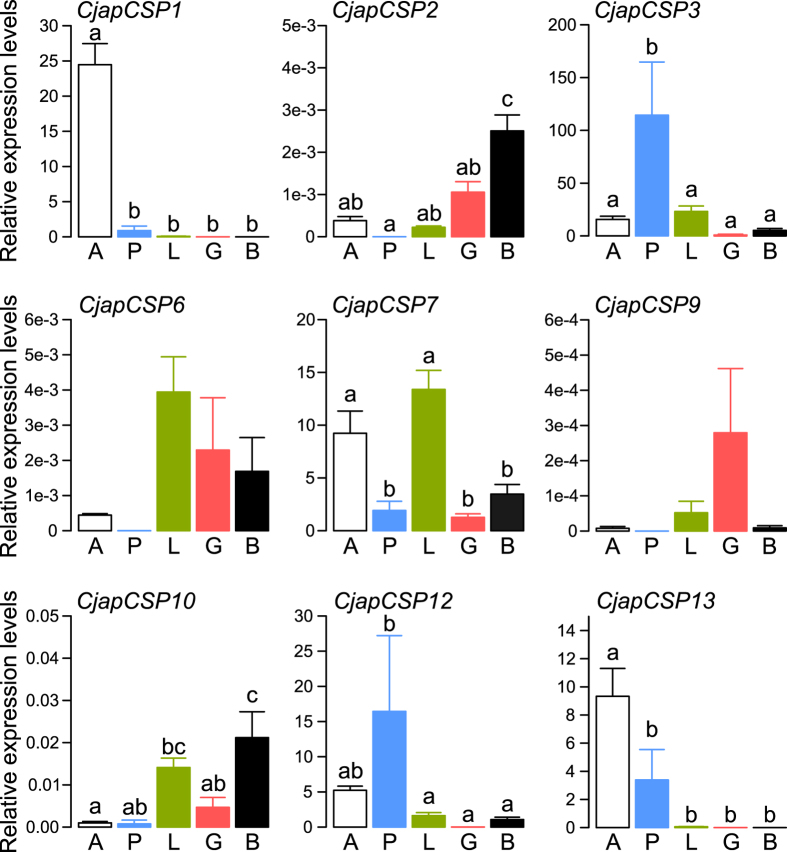
Relative mRNA levels of chemosensory protein (CSP) genes in various tissues of *Camponotus japonicus* workers. Expression levels are normalised to that of *rpl18*. Significant differences are indicated by different letters (Tukey’s HSD test, p < 0.05). A: antennae (N = 5 biological replicates), P: palps (N = 3 biological replicates), L: legs (N = 5 biological replicates), G: gut (N = 5 biological replicates), B: rest of body (N = 5 biological replicates).

We performed qRT-PCR analysis of the 12 CSP genes in the antennae, palps, legs, gut, and the rest of the body of *C. japonicus* workers from three independent colonies. Among the 12 annotated CSP genes, *CjapCSP4*, *5*, and *8* were omitted from further quantitative analysis, because they did not indicate linearity in the PCR amplification curves under our experimental conditions. Except for *CjapCSP9*, all the CSP genes were differentially expressed among tissues (Fig. 3; ANOVA, p < 0.05). Regarding the conserved CSP genes, *CjapCSP1* was highly expressed in the antennae (post-hoc Tukey’s HSD test, p < 0.05); *CjapCSP2* was highly expressed in the body parts (p < 0.05); *CjapCSP3* was highly expressed in the palps; and *CjapCSP7* was highly expressed in the antennae and legs (p < 0.05). Although the post-hoc test was non-significant, *CjapCSP6* was less expressed in the antennae and palps. Regarding the ant-specific CSP genes, *CjapCSP10* was highly expressed in the non-chemosensory organs (p < 0.05) and both *CjapCSP12* and *13* were highly expressed in the antennae and palps (p < 0.05).

### Localization of CSP transcripts in worker antennae

**Figure 4:**
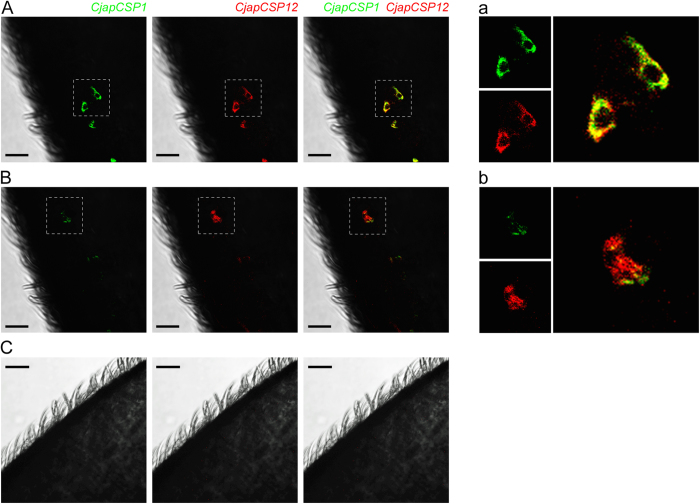
Two-colour fluorescent *in situ* hybridization of *CjapCSP1* and *CjapCSP12* transcripts in *Camponotus japonicus* worker antennae. Single optical plane of antennal surfaces visualised by fluorescent dies with *CjapCSP1* (green) and *CjapCSP12* (red) anti-sense probes (**A**,**B**) or sense probes (**C**). (**A**) Cells labelled with both *CjapCSP1* and *CjapCSP12* probes. Yellow colour indicates co-labelling with two probes. (**B**) Cells mainly labelled with *CjapCSP12* anti-sense probes. (**a**,**b**) Higher magnification of the areas boxed in (**A**,**B**), respectively. Scale bars indicate 20 μm.

**Figure 5:**
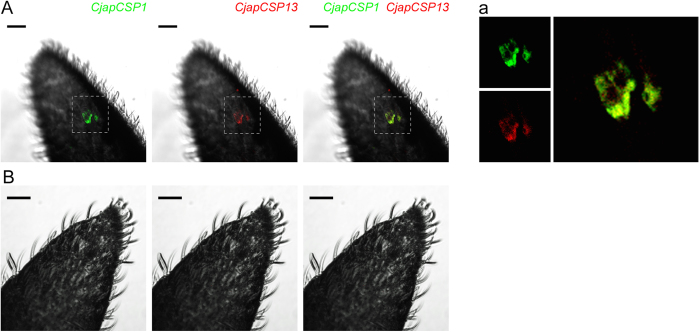
Two-colour fluorescent *in situ* hybridization of *CjapCSP1* and *CjapCSP13* transcripts in *Camponotus japonicus* worker antennae. Single optical plane of antennal surfaces were visualised by fluorescent dies with *CjapCSP1* (green) and *CjapCSP13* (red) anti-sense probes (**A**) or sense probes (**B**). (**A**) Cells labelled with both *CjapCSP1* and *CjapCSP13* probes. Yellow colour indicates co-labelling with two probes. (**a**) Higher magnification of the areas boxed in (**A**). Scale bars indicate 20 μm.

Two-colour *in situ* hybridization was conducted to identify the spatial localization of CSP genes in worker antennae. We selected three CSP genes (*CjapCSP1*, *12*, and *13*) that were highly expressed in the chemosensory organs, including worker antennae. Using pairs of differentially labelled *CjapCSP*-specific probes, antenna cells containing transcripts for each *CjapCSP* were visualised by green or red fluorescence. We paired *CjapCSP12* or *CjapCSP13* with *CjapCSP1*, which are expressed in the antennal sensilla basiconica^16^. The *CjapCSP1*-*CjapCSP12* pairing (17 out of 36 samples observed) showed that about 90% of the labelled cells were overlapped (Fig. 4A), but about 10% cells were mainly labelled by *CjapCSP12* probes (Fig. 4B). The *CjapCSP1*-*CjapCSP13* pairing (19 out of 31 samples observed) showed that the labelled cells by both probes were overlapped (Fig. 5A). These results indicated a co-expression of *CjapCSP*s in worker antennae. Control experiments with labelled-sense probes for the *CjapCSP1*-*CjapCSP12* (N = 30) and *CjapCSP1*-*CjapCSP13* (N = 31) pairings did not detect any specific signals (Figs 4C and 5B).

## Discussion

In this study, we demonstrated that some CSP genes (*CjapCSP1*, *3*, *7*, *12*, and *13*) were highly expressed in the chemosensory organs of workers (Fig. 3), and they showed differential expression among the antennae of the three castes, except for *CjapCSP7* (Fig. 2). In addition, *CjapCSP4* was highly expressed in the antennae of the three castes (Fig. 2). These results suggested that *CjapCSP1*, *3*, *4*, *7*, *12* and *13* were mainly involved in peripheral chemosensory events. Our phylogenetic analysis supported the basic model of CSP gene evolution reported by Kulmuni *et al*.^8^ and McKenzie *et al*.^24^ (Fig. 1). We also showed that, in the expanded group of ant-specific CSP genes (*CjapCSP10*–*13*), *CjapCSP12* and *CjapCSP13* were mainly expressed in the chemosensory organs (Figs 2 and 3). The branch-site tests for positive selection were non-significant for all *Camponotus* CSP gene pairs, except for the ant-specific CSPs that were significant, indicating a positive selection in this group (Table 1). Furthermore, *Camponotus CSP9, 12*, *13* had a higher dN/dS ratio than the other CSP genes (Table 2). Although we cannot eliminate the possibility that the estimated high dN/dS ratios were due to low dS rather than high dN, it is plausible that that they have been under positive selection and the functional differentiation of *CjapCSP12* and *13* is involved in chemosensory events related to ant-specific functions.

Some genes belonging to the CSP gene family have been reported not to be involved in chemoreception, but to have another function^27^,^28^,^29^. For example, in honeybees, *AmelCSP5* is expressed in the queen ovaries and eggs, but not in the antennae^27^, and RNA interference inhibition of *AmelCSP5* suggests its participation in embryonic development^29^. In addition to *CjapCSP5*, *CjapCSP2*, *CjapCSP6*, and *CjapCSP9* and even the ant-specific *CjapCSP10* were mainly expressed in the non-chemosensory organs; however, owing to their low relative expression levels in qRT-PCR (Fig. 3), their biological relevance should be carefully considered. Non-chemosensory functions are also observed in the OBP family. In *Solenopsis invicta*, the pheromone-binding protein Gp9 is a hemolymph protein probably involved in the transport of hormonal signals^30^. Gp9 genes form a supergene cluster that results in two distinct forms of social phenotypes^31^. Therefore, it would be interesting to investigate the functional aspect of expanded ant-specific CSPs, even though they are non-chemosensory related.

Although CSPs bind a wide range of chemical compounds, the binding spectra are clearly different between CSPs^32^,^33^. Ligand-binding properties of CSPs depend on various traits, such as binding-pocket size and surface charges^23^. Ant CSP8 and CSP1 are known to have smaller binding pockets than typical CSPs, which suggests that they have different binding spectra compared to typical CSPs^23^. CjapCSP1 is known to have a wide binding spectrum for cuticular hydrocarbon components that cause the colony-specific odour of workers^16^. Binding assays suggest that CjapCSP1 introduces the hydrocarbon mixture into receptor lymph in the same mixing ratio as they were originally presented on the body surface cuticle. CjapCSP12 and CjapCSP13 are duplicated from the CjapCSP1 lineage (Fig. 1), and they are co-expressed in some chemosensilla. Therefore, it would be interesting to investigate whether CjapCSP12 and CjapCSP13 are also involved in transporting hydrocarbon signals.

The co-expression of carrier proteins is known in some OBPs^34^,^35^,^36^,^37^. The complex expression pattern of OBPs suggests that the binding proteins not only act as ligand transporters, but also contribute to odour recognition^12^,^35^,^37^. Among the ant-specific CSPs, *CjapCSP12* and *13* are co-expressed with *CjapCSP1*. In *C. japonicus*, the sensilla basiconica filled with CjapCSP1 in its sensillar cavity accommodates about 130 receptor neurons^16^ and is involved in the discrimination of nestmate and non-nestmate hydrocarbon signatures^16^. In addition to nestmate and non-nestmate discrimination^18^, cuticular hydrocarbon signatures are used for various recognition processes in ants, such as tasks^19^, fertility^20^, individuals^21^, mutualistic partners^38^, as well as for the queen substances that suppress the ovarian development in the workers^22^. Therefore, cuticular hydrocarbons are important chemical signals in ant societies. It is possible that several carrier proteins are involved in the transportation and recognition of hydrocarbon compounds. Although the contribution of binding proteins to ligand-selectivity in the peripheral chemosensory systems remains unclear, co-expressing CSPs might work cooperatively in the sensillar lymphs.

RNA-seq analysis shows that *CjapCSP3* and *CjapCSP12* are highly expressed in male antennae (Fig. 2). Furthermore, *CjapCSP3* and *CjapCSP12* are also highly expressed in the maxillary and/or labial palps of workers (Fig. 3), and they might be involved in gustatory perception by binding lipophilic chemicals in these chemosensory organs. The palps are known as gustatory rather than olfactory organs in ants^39^, and their ligands are generally hydrophilic, such as sugars and amino acids. The expression of lipophilic ligand-binding proteins in the gustatory organs is known in some insects^40^, however, and these proteins are involved in the avoidance of noxious compounds^41^. Some hydrocarbon compounds are also known as repellents for some insects^42^ and are detected by gustatory receptor neurons^43^.

Overall, our results indicated that two ant-specific CSPs (*CjapCSP12* and *CjapCSP13*) are highly expressed in the chemosensory organs, and these genes tend to show higher dN/dS ratios than others. These CSPs are co-expressed with *CjapCSP1* around the base of antennal chemosensilla in workers. Because CjapCSP1 is known to bind pheromonal cuticular hydrocarbons, it is possible that these two ant-specific CSPs have a role in chemical communication. Further expression studies and ligand-binding assays using recombinant proteins may reveal any uncharacterized functions of the CSP gene family in ants.

## Materials and Methods

### RNA isolation

RNA was isolated from the three different castes (males, alate queens, and workers) of *C. japonicus* obtained from 1–4 colonies found at the campus of Kobe University, Japan. Males and queens were collected during the nuptial flight in spring and used for RNA extraction within a few days. Workers were collected in autumn and kept in the laboratory for about three months before RNA extraction. In order to investigate gene expression levels in the antenna of each caste, the antennal tissue was separately treated with the other parts of the body. Both antennae were isolated from about 100 individuals using fine tweezers and immediately frozen with liquid nitrogen and crushed in a hand mortar, while the body without the antennae was also frozen with liquid nitrogen. Total RNA of each sample was extracted using ISOGEN (Nippon gene, Tokyo, Japan), according to the manufacturer’s standard protocol. RNA samples were treated with DNase (RNase-free DNase Set; Qiagen, Hilden, Germany) and then purified with RNeasy Mini Kit (Qiagen).

We obtained 22 RNA samples: 9 antennal (three biological replicates for each caste) and 13 body samples (6, 3, and 4 for males, queens, and workers, respectively). One of 3 antennal samples of workers was divided into 4 parts, and thus 25 samples in total were used for further cDNA library generation procedure.

### RNA sequencing

cDNA libraries were generated from purified RNA (0.5 μg of each sample) using TruSeq RNA Sample Preparation Kit v2 (Illumina Inc., San Diego, CA) according to the manufacturer’s protocol (Low Throughput Protocol), except that all reactions were carried out at half scale. The fragmentation of mRNA was performed for 4 min and PCR cycles ranged from 8 to 12, depending on the sample. In total, 21 multiplexed libraries were sequenced in two lanes using Hiseq2000 (Illumina Inc., San Diego, CA) with 101-bp paired-end readings and 4 libraries in one lane using HiSeq platform (Illumina Inc., San Diego, CA). Raw data processing, base calling, and quality control were performed according to the manufacturer’s standard protocol using RTA, OLB, and CASAVA software (Illumina Inc., San Diego, CA). Sequence quality was inspected by FastQC (http://www.bioinformatics.bbsrc.ac.uk/projects/fastqc/).

The reads were cleaned up with cutadapt^44^. Low-quality ends (<QV30) and adapter sequences were trimmed, while reads shorter than 50 bp were discarded. To build a comprehensive set of reference transcript sequences, we used all paired-end reads derived from the antenna and body of all three castes. Cleaned reads from 21 libraries with paired-end reading were assembled together using Trinity^26^, an RNA-seq *de-novo* assembler, in the paired-end mode with the ‘−min_kmer_cov = 2’ option.

ORFs were extracted from the Trinity contigs using TransDecorder, which is included in the Trinity suite. The predicted ORF sequences were grouped using CD-HIT-EST^45^ with a minimum identity of 97%, and finally a non-redundant coding sequence set (CDS reference) was obtained.

For differential expression analysis, we used library reads from male (N = 3 biological replicates), alate queen (N = 3 biological replicates), and worker (N = 3 biological replicates) antennae. From four technical replicate worker antennal samples, we chose one sample that showed an average expression pattern in the nonmetric multidimensional scaling (nMDS) plot of the whole transcriptome data. We mapped Read-1 sequences for each a library to the CDS reference using Bowtie2 software with the ‘-local,–all’^46^ parameter, and then transcript abundance was estimated using eXpress^47^. To adjust library sizes and skewed expression of transcripts, the estimated abundance values were normalised using the Trimmed Mean of M-value (TMM) normalization method^48^, and differential expression analysis was conducted using edgeR package^48^.

### CSP gene identification

We first used tBLASTx to search the Trinity assembly sequences of *C. japonicus* for CSP candidates using nucleotide sequences of other insect species (*Drosophila melanogaster*^49^, *A. mellifera*^27^, and 7 different ant species^8^) as queries with an e-value cutoff of 1.0E–3. We also ran a HMM search using the OS-D superfamily (pfam03392) as a query. Although there are several naming systems of ant CSPs, we adopted the most recent naming system described by McKenzie *et al*.^24^. To confirm the assembled nucleotide sequences, cDNA of 7 *CjapCSP*s were subcloned and sequenced. Total RNA of worker antennae (for *CjapCSP1*, *CjapCSP3*, *CjapCSP4*, *CjapCSP7*, *CjapCSP12*, and *CjapCSP13*) or heads (for *CjapCSP2*) was extracted as described above, and each cDNA was synthesized by SuperScript II Reverse transcriptase (Invitrogen, Carlsbad, CA). Coding sequences of 7 *CjapCSP*s were obtained through PCR amplification with gene-specific primers (Supplemental Table S1) and Takara Ex Taq (Takara, Tokyo, Japan). PCR products were subcloned into the pGEX-4T-2 vector (GE Healthcare, Piscataway, NJ), and nucleotide sequences of each fragment were determined with Big Dye Terminator v3.1 Cycle Sequencing Kit using Model3100 Genetic Analyzer (Applied Biosystems, Foster City, CA). Potential signal peptides were predicted by SignalP (http://www.cbs.dtu.dk/services/SignalP/)^50^. We used SWISS-MODEL (http://swissmodel.expasy.org)^51^ to model the structure of *Camponotus* CSPs using MbraCSPA6 (PDB-ID:1KX9) from *Mamestra brassicae*^14^ as reference.

### Alignment and phylogenetic trees

We produced an alignment with the G-INS-i strategy of MAFFT^52^ using CSP sequences of *D. melanogaster*^49^, *A. mellifera*^27^, *P. canadensis*^24^, and 9 different ant species^23^,^24^ including *C. japonicus*. This alignment was used to produce a maximum likelihood phylogenetic tree using RAxML (http://sco.h-its.org/exelixis/software.html)^53^ with 100 bootstrap replicates.

### Molecular evolutionary analyses

We obtained codon-level alignment of insect CSPs using PRANK software (option-‘once’)^54^, and the maximum likelihood tree produced by RAxML was used as reference. For branch-site test, ‘alternative model A’ and ‘null model A’ were constructed for each pairwise branch of *Camponotus* CSPs and the expanded ant-specific CSPs as foreground branches using the codeml program in PAML^55^. The p-values obtained from likelihood ratio tests were corrected for multiple comparisons using qvalue package^56^ in R software^57^.

For calculating the pairwise maximum likelihood (ML) estimation of the dN/dS ratio, we preliminarily aligned the 25 *Camponotus* CSP sequences with MAFFT G-INS-i strategy, and an initial tree was built using RAxML with 100 bootstrap replicates. This phylogenetic tree was used to obtain a codon-level alignment of the 25 *Camponotus* CSP nucleotide sequences using PRANK software (option-‘once’)^54^, while the ambiguous sections of the alignment for each orthologous pair of *Camponotus* CSP genes were removed using trimAl (option-‘gappyout’)^58^. For the 12 orthologous CSP gene pairs of *C. floridanus* and *C. japonicus*, we used the program codeml in PAML^55^ to calculate the ML estimation of the pairwise dN/dS ratio for each orthologous *Camponotus* CSP gene.

### qRT-PCR analysis

Tissues of *C. japonicus* workers were dissected using micro scissors under a stereomicroscope. In each experiment, the antennae and legs (tibia and tarsus) were cut from 25 ants, while palps (maxillary and labial) were collected from 200 ants. Gut tissues were dissected from 10 ants after enteral contents were washed away in a phosphate-buffered saline solution (128 mM NaCl, 5 mM KCl, 2 mM MgCl_2_, 1 mM Na_2_HPO_4_, 0.34 mM KH_2_PO_4_, 1.83 mM CaCl_2_, and 25 mM glucose). The remaining parts of the body were also collected from 10 ants. Each tissue was immediately frozen with liquid nitrogen and crushed in a hand mortar. Total RNA was extracted with Sepasol-RNA I Super G (Nacalai tesque, Kyoto, Japan). The degradation of genomic DNA and synthesis of cDNA were performed with ReverTra Ace qPCR RT Master Mix with gDNA Remover (Toyobo, Osaka, Japan) according to the manufacturer’s protocol. Primer sequences are listed in Supplemental Table S2. qRT-PCR was performed with Thunderbird SYBR qPCR Mix (Toyobo, Osaka, Japan) using Thermal Cycler Dice Real Time System II (Takara, Tokyo, Japan).

Reference genes were selected according to a recent report on *C. floridanus*^59^, an ant species closely related to *C. japonicus*. Briefly, we selected elongation factor 1 (*ef1*), 60S ribosomal protein L32 (*rpl32*), 60S ribosomal protein L18 (*rpl18*), and glyceraldehyde-3-phosphate dehydrogenase (*gapdh*) as candidate reference genes, and performed tBLASTx search for each homologous gene from the *C. japonicus* RNA-seq data. These 4 genes have been commonly used as references, because they are expressed at similar levels in several body parts of *C. floridanus*^59^. The suitability of these genes as references in our experimental conditions was determined using BestKeeper software^60^. Among these 4 genes, *rpl18* showed the lowest standard deviation value, indicating that it was the most suitable reference gene. Expression levels of CSP genes were normalised with that of *rpl18* using the ∆Ct method. For each CSP gene, the homogeneity of variance was analysed with Leven’s test, and then the data were statistically analysed by one-way analysis of variance (ANOVA) in conjunction with post-hoc Tukey’s HSD test. If the null hypothesis of equal variances was rejected (*CjapCSP3*, *7*, *8*, and *12*), one-way ANOVA with Welch’s correction for non-homogeneity was applied. All statistical analyses were conducted using R software^57^.

### *In situ* hybridization

Digoxigenin (DIG)-labelled and biotion-labelled RNA probes for *in situ* hybridization were synthesized by *in vitro* transcription using DIG RNA Labelling Mix (Roche, Basel, Switzerland) and Biotin RNA Labelling Mix (Roche, Basel, Switzerland). The primers that used to synthesize RNA probes targeting the exon regions of each *CjapCSP* are listed in Supplemental Table S3.

Two-colour fluorescent *in situ* hybridization was performed as described by Qiao *et al*.^61^ with minor modifications. Antennae dissected from cold anesthetized workers were fixed in a solution composed of 4% paraformaldehyde, 0.1 M Na_2_CO_3_, 0.03% Triton X-100 (pH 9.5) for 20–24 h at 6 °C. After a brief wash in PB with 0.03% Triton X-100 at room temperature, one side of the cuticle on the flagellum was cut off with a thin blade under a binocular inspection microscope. The sliced antennae were incubated in 0.2 N HCl and 0.03% Triton X-100 for 10 min, washed for 1 min in PB containing 1% Triton X-100, and pre-hybridised at 55 °C for at least 6 h in an *in situ* hybridization solution (50% formamide, 5 × saline-sodium citrate (SSC), 1 × Denhardt’s solution, 50 μg ml^−1^ yeast RNA, 1% Tween 20, 0.1% CHAPS, and 5 mM EDTA [pH 8.0]). Antennae were further hybridized with labelled anti-sense RNA probes at 55 °C for at least 48 h. Antennae were washed four times for 15 min each in 0.1 × SSC and 0.03% Triton X-100 at 60 °C and then incubated in 1% blocking reagent (Roche, Basel, Switzerland) for 5 h at 6 °C. For the detection of DIG- and biotin-labelled RNA probes, samples were reacted with an anti-DIG mouse IgG1 antibody (Fluorescent Antibody Enhancer Set; Roche, Basel, Switzerland) and streptavidin conjugated with horseradish peroxidase (HRP) (PerkinElmer, Salem, MA). Samples were incubated for at least 48 h at 6 °C, and antennae were washed three times in PB with 0.2% Tween 20 for 10 min each at room temperature. The detection of DIG-labelled probes was performed using Fluorescent Antibody Enhancer Set (Roche, Basel, Switzerland) according to the manufacturer’s protocol. After three 5-min washes in PB with 0.2% Tween 20, the biotin-labelled probes were visualised using the TSA kit/HNPP Fluorescent Detection Set (PerkinElmer, Salem, MA). After washing three times for 5 min each in PB with 0.2% Tween 20, antennae were mounted in Mowiol solution (10% polyvinyl alcohol 4–88 and 20% glycerol in PB). Confocal laser scanning microscopic observations were performed using FV1000 (Olympus, Tokyo, Japan).

## Additional Information

**Accession codes:** DDBJ accession numbers for RNAseq data is DRA002913 and for *CjapCSP*s are LC028282–LC028288. 

**How to cite this article**: Hojo, M. K. *et al*. Antennal RNA-sequencing analysis reveals evolutionary aspects of chemosensory proteins in the carpenter ant, *Camponotus japonicus*. *Sci. Rep*. **5**, 13541; doi: 10.1038/srep13541 (2015).

## Supplementary Material

Supplementary Information

Supplementary DataSet 1

Supplementary DataSet 2

## Figures and Tables

**Table 1:** Branch-site tests for positive selection of the foreground branch of expanded ant-specific chemosensory proteins (CSPs) and each *Camponotus* CSP branch.

	-Ln (likelihood)		LRT		
	Model_A Alt	Model_A Null	df	p-value	q-value
Ant expansion	33568.1	33572.6	1	0.002	0.037
*CSP1*	33572.7	33572.7	1	1	1
*CSP2*	33572.7	33572.7	1	0.984	1
*CSP3*	33572.3	33572.6	1	0.421	1
*CSP4*	33572.7	33572.7	1	1	1
*CSP5*	33569.7	33570.6	1	0.180	0.586
*CSP6*	33572.7	33572.7	1	1	1
*CSP7*	33572.7	33572.7	1	1	1
*CSP8*	33571.6	33571.6	1	1	1
*CSP9*	33572.5	33572.7	1	0.561	1
*CSP10*	33569.4	33571.8	1	0.029	0.191
*CSP12*	33571.1	33572.7	1	0.076	0.332
*CSP13*	33572.6	33572.6	1	0.775	1

**Table 2:** The ratio of divergence at non-synonymous and synonymous sites (dN/dS) for each orthologous pair of *Camponotus* chemosensory protein (CSP) genes.

Orthologous pairs	dN	dS	dN/dS
*CSP1*	0.01	0.09	0.20
*CSP2*	0.01	0.02	0.45
*CSP3*	0.07	0.10	0.66
*CSP4*	0.00	0.06	0.12
*CSP5*	0.04	0.08	0.46
*CSP6*	0.00	0.03	0.00
*CSP7*	0.04	0.07	0.61
*CSP8*	0.03	0.10	0.29
*CSP9*	0.09	0.01	7.75
*CSP10*	0.11	0.18	0.65
*CSP12*	0.08	0.05	1.41
*CSP13*	0.06	0.06	1.05
