# Supplementary material for: Antennal RNA-sequencing analysis reveals evolutionary aspects of chemosensory proteins in the carpenter ant, Camponotus japonicus
Source: Sci Rep. 2015 Aug 27;5:13541. doi: 10.1038/srep13541 (PMC4550911; doi:10.1038/srep13541)
Supplement: Supplementary Information [file srep13541-s1.pdf]

Supporting Information

Antennal RNA-sequencing analysis reveals evolutionary aspects of chemosensory proteins in the carpenter ant *Camponotus japonicus*

Masaru K. Hojo[1]*, Kenichi Ishii[2]*, Midori Sakura[1], Katsushi Yamaguchi[3], Shuji Shigenobu[3,4], Mamiko Ozaki[1]

[1]Department of Biology, Graduate School of Science, Kobe University, Kobe, Japan

[2]Department of Biological Sciences, Graduate School of Science, The University of Tokyo, Tokyo, Japan

[3]NIBB Core Research Facilities, National Institute for Basic Biology, National Institutes of Natural Sciences, Okazaki, Japan

[4]Department of Basic Biology, School of Life Science, Graduate University for Advanced Studies, Okazaki, Japan

*Equal contribution

Supplemental Tables

Supplemental Table S1. Nucleotide sequences of primers used for cloning of *CjapCSPs.*

Lower-case letters indicate additional sequences for restriction enzyme recognition.

Gene	Forward primer (5'-3')	Reverse primer (5'-3')
CjapCSP1	aaggatccGAGGAAATGTACTCGGACATG	aactcgagTCAACGGGTAAAGGGGATG
CjapCSP2	aaggatccGCCGAATCGGACAATAGCG	aactcgagTTATGAAACTCCAGCGTATTGTTG
CjapCSP3	aaggatccCGGCCACAAGACCATTATAC	aactcgagTTAATTTTCTTTCTTCAAGGCTCGATC
CjapCSP4	aaggatccGAGGACGTGCAGTATACTAC	aactcgagTCAATCCAATCTGCCTCCTTC
CjapCSP7	aaggatccGACGATAAGTACACGACCAAG	aactcgagTCACGCTTTTATGTTGCGTTTCTC
CjapCSP12	aaggatccGAGGAAGAGAAATACGAGGATAA	aactcgagTTATTCTTTAGCTTTTTTCTTCATATCTTCTAAA
CjapCSP13	aaggatccGAAGAACTTTATAGCGATCAGTACG	aactcgagTTATTGACCAGCATTCTTCTTTTTCATATCTTC

Supplemental Table S2. Nucleotide sequences of primers used for RT-PCR.

Gene	Forward primer (5'-3')	Reverse primer (5'-3')
Elongation factor 1 (*ef1*)	CCCTTCGTCTTCCCCTTCA	ACCATACCGGGTTTCAGCA C
60S ribosomal protein L32 (*rpl32*)	CCAACAGGCTTCCGTAAAG TTC	TCTCTGCACAAAACTTGCGA TT
60S ribosomal protein L18 (*rpl18*)	AAGAAACCTGGACGGGAG AA	CGAGCTCTTTCGGTTACACG
Glyceraldehyde-3-phos phate dehydrogenase (*gapdh*)	CATGACGACTGTACATGCG ATT	GCAGCGGGAATAATGTTTT G
CjapCSP2	ATTCGCGACGAAATGTCGA A	CTGGGGACGATTCTCGGTGT
CjapCSP2	GCGACAAGCGTTACTTGAC C	CAGGAGCCTCTCAAAACCA A
CjapCSP3	CAAATGCCCCTCTGAAGCT C	CGCCTTTCTCGCAATTTCTT
CjapCSP4	AGATGCGGCGGAACTCAA	CGCTGTTCGCAATTTAAACG A
CjapCSP5	CAGGGGATTGCGATGGATT T	CGGGGTACAACGACGACAT TT
CjapCSP6	GGTGTTTGTTGGACGAAGG A	TTCTGCCGTAGCCTTTTGTT T
CjapCSP7	TGCCTGCTGGATAAGGGAA A	GCATTTGCTGCACTCGGTCT
CjapCSP8	CCGATGCACGCAGTCTTAA A	TGCCTATTTTTCTTGCTCCTT G
CjapCSP9	TACGGGATCTGCACGCTTC T	TCGGTAATCGCATTGAGAA TGT
CjapCSP10	GTGCAAGACAGCTGATCAA AGA	ACCAATCGAGTACGATTTCC AA
CjapCSP12	GTTTCATGGCAACAGGACC A	TGCACTTAGTTTGAAAAGCC TCAC
CjapCSP13	CCATGTCTAACAGCAGAAG CAAA	TCAACGATCTTCTGCCATTT GT

Supplemental Table S3. Nucleotide sequences of primers used for *in situ* hybridization

Lower-case letters indicate additional sequences for restriction enzyme recognition. Note that the same primers listed in gene cloning (Supplemental Table S1) were used for *CjapCSP12* and *CjapCSP13*.

Gene	Forward primer (5'-3')	Reverse primer (5'-3')
CjapCSP1	GGACATGTTCGATCACATCAA	CCTCGAAAACTACACATGATGC
CjapCSP12	aaggatccGAGGAAGAGAAATAC GAGGATAA	aactcgagTTATTCTTTAGCTTTTTTC TTCATATCTTCTAAA
CjapCSP13	aaggatccGAAGAACTTTATAGC GATCAGTACG	aactcgagTTATTGACCAGCATTCTT CTTTTTCATATCTTC

Supplemental Dataset

Supplemental dataset S1. Nucleotide sequences of twelve annotated CjapCSPs

Supplemental dataset S2. Amino acid sequences of twelve annotated CjapCSPs
