# Supplementary material for: Antennal RNA-sequencing analysis reveals evolutionary aspects of chemosensory proteins in the carpenter ant, Camponotus japonicus
Source: Sci Rep. 2015 Aug 27;5:13541. doi: 10.1038/srep13541 (PMC4550911; doi:10.1038/srep13541)
Supplement: Supplementary DataSet 1 [file srep13541-s2.doc]

>CjapCSP1

ATGGATAAATCGAGTCTCTGCTTGCTGGCCCTCGGCGTGCTGGCGGCCGTCATCGCCGAG

GAAATGTACTCGGACATGTTCGATCACATCAATCCCGATGATATCTTGCCCAACGATGAG

CTACGAAATCAGTATTATAACTGCTTCATGGATACTGGCCCATGCGTGACAGAGGATCAG

AAATATTTCAAGGAGCATGCTGCCGAGGCATTCGCGACGAAATGTCGAAAATGCACGGAA

GTGCAAAAGAAGAACGTGGAGAAGATAGTCGTGTGGTACACCGAGAATCGTCCCCAGGAG

TGGCAGGCGATGGTGCAAAAACTCATGGACGATGCGAAGAAGTTGAACATCCCCTTTACC

CGTTGA

>CjapCSP2

ATGGCTCTGACAATCAAGTTCCTTATCCTCGTCTGCGCCCTGTTCACCGCGACGATGGCA

GCCGAATCGGACAATAGCGAGGGACAGCAATCCGGCAGATCGCGGGTTTCTGATGAACAA

CTGAACATAGCATTGAGCGACAAGCGTTACTTGACCAGACAGCTTAAGTGCGCCCTGGGA

GAAGCGCCATGCGATCCCGTTGGGCGACGCTTGAAAAGCTTGGTGCCATTGGTTTTGAGA

GGCTCCTGCCCGCAATGCAGTCCGGAAGAGACACGTCAGATCAAGAAGGTCCTTTCTCAC

ATTCAGCGATCCTTTCCGAAGGAGTGGAGCAGGATAGTGCAACAATACGCTGGAGTTTCA

TAA

>CjapCSP3

ATGAAATTTGCACTGGTGTGTCTATTCGCTATTAGCACTATCGTCTGTGTGTATGGACGG

CCACAAGACCATTATACCGATAAATTTGACAATATAGATGTAGACCAAATCTTAAACAAT

GACCGATTATTGAAACGATACGTAGATTGCTTGTTGGAAAGGTCCCATGTCAAATGCCCC

TCTGAAGCTCTTGAACTAAAGAAAGTTTTAGCCGATGCAATGGCAACTGACTGTGCCAAG

TGCACGGATCGACAGAAAGAAATTGCGAGAAAGGCGTTAGACTTCCTAATAATAAACAAA

ACAGATATGTGGAACGATCTGAAGTCTAAGTACGATCCGGAAGAGAAATACGCGAAAAAG

TATGAAGATCGAGCCTTGAAGAAAGAAAATTAA

>CjapCSP4

ATGAAGCATCTAGTCGTCGCTCTTATCACCGCCCTTTCGTTTTCCGTCGTTCTCGCAGAG

GACGTGCAGTATACTACGAAATACGACAATATAGATGTGGACGCGGTGATAAACAGCGAG

AGGCTGTTGAATGGATACGTGGGTTGTCTGCTCGATCGAACTCCCTGCACCCCAGATGCG

GCGGAACTCAAGAAAAATCTACCGGATGCGTTAGAACACGACTGCGCCGGATGCAGCGAG

ATGCAAAAGAATGCAGCCGACAAAATCTCTCATCATCTGATCGACAATAAGCCGGACGAT

TGGAGACTTTTGGAGGACAAGTATGACCCTACCGGGGCATATCGACGACGTTACTTGGAA

AACAAATCTCACGAAGGAGGCAGATTGGATTGA

>CjapCSP5

ATGAATAAGCAGATTATTATTTTAATTATCATCGGCTCAGGACTTGCCGTTTTTTGCCAA

GCGCAAGATATTTCGTCATATTTAACAGATAAACGTTTCATCGATAAGGAACTTCATTGC

TTACTCGAGACAGGGGATTGCGATGGATTTGGAAAACAAATAAAACGTGTATTACCCGTA

GTACTCAAGGATAAATGTCGTCGTTGTACCCCGCAGCAGAAAGCAAATCTACATAAATTG

ATACAATTTCTACAATCGCGATATCCCACGCAATGGCACACGATCGAAGAAATGTATTCT

TCACCAACTTTTCAATGA

>CjapCSP6

ATGAAGAAATATTTGTTGATCTCGTTGGCCTCGTTGATGATTCTGGTTGTTGCCACTGAG

AAATATACCGGAAAATACGATGACGTGGATGTGGATAAGATTCTTCAAAATAATCGCGTC

CTCAATAATTACATCCGGTGTTTGTTGGACGAAGGACCCTGCACTGCCGAAGGTCGCGAA

TTAAGAAAGACTTTACCGGATGCTTTATCGAGCAGTTGCAGCAAGTGCAATGATAAACAA

AAGGCTACGGCAGAAAAAGTGATAAATCATCTCAAAACAAAACGATCCAAGGATTGGGAT

CGTCTTATTGCCAAATACGATCCTCGTGGCGAATATAAAAAACGTTATGAGCAATTGTAG

>CjapCSP7

ATGAAGGTCCTGGCTCTACTCCTCATCGCTGTAGCTTGCGCTTTGGCGGACGATAAGTAC

ACGACCAAGTTCGACAATATCGACGTAGACGCTATTCTGAAGAGCGACCGCCTGCTCAAG

AACTACGTAAATTGCCTGCTGGATAAGGGAAACTGCACGCCCGATGGCAAGGAACTCAAA

GAACACCTTCCGGACGCATTGGAGACCGAGTGCAGCAAATGCAGCGAGAAGCAAAGAACC

GGCACCGAGAAGGTCATCCGGTTTTTAGTGAATAAGAAACCAGAAACATGGGAGCAACTT

AAGAAGAAGTACGATCCTAATGGTGAATACTCTCGACGGTACGAGGATGAGGCTGAGAAA

CGCAACATAAAAGCGTGA

>CjapCSP8

ATGAAACTCCCATTTCTATTATTGCTGTCCAGTTTCGTATTCTGTGGACTCGTTTCAGGG

ACGGAAAATTATACGGATATACATGACAATGTAGATATAGATGCAATTCTTAATAGTGAT

CGTCTTTTAAAGCAATATATGGATTGCATTCTAGAAAAAGGTTCCTGCACGGCCGATGCA

CGCAGTCTTAAACGTATACTTCCAGAGGCGGTAGCTACAATTTGCGAAAAATGCAATTTA

AAACAAAGACAAGGAGCAAGAAAAATAGGCAATCATTTGAAAAAGTATAAACCAGAGCTC

TGGACGATATTTCTTGAAAAATATGATCCAAACAAAGAGTATATTGAAAATTTCGAACAA

TTTCTAGCACAAGTAGAAGAATAA

>CjapCSP9

ATGACTAAATTAGTTAGTTGCACATTTGCTTGTCTCGTCATGACATTGGCGGTGCTAATT

GCGCATGCAGAAGATGAAAAATATTCGAGCAAATACGATCATATCGATATTAACGAGGTG

TTGGCTAATTCTCGCTTGAGGAATCAATATGTCAGATGTTTAATAAATATTTCTCCCTGT

ACTACGGGATCTGCACGCTTCTTAAAAGATATACAAGGGGAAGCATTTGTTACAAAGTGT

AAAAAATGTACGGATAAGCAAATATACATTCTCAATGCGATTACCGATTGGTTTACGAAA

AATGAACCTGAAACTTGGAATCGTATGGTGCAAGTAGCAGTCGAGGAAGCAAAAAGGAAA

AATGCATAA

>CjapCSP10

ATGGCTCGACCAAGTTACATCGTAGCGATCGTTGTCATTGCATTAACGTGTGTTCTCGCA

GAGGAACTCTATTCCAGTCGATTCGATGACGTCGATGTCCGCGCAATTTTTAATAATGCT

AAATTGCGAAACCAATATTATAATTGTTTTATGGATTTATCACCGTGCAAGACAGCTGAT

CAAAGATTCTTTAAAGGGATTTTTAGTGAAGCTTTACAATCTGGATGTAAGAGATGTACT

GAAAAGCAAAAAGAAAATTTGGAAATCGTACTCGATTGGTATACAATCAATGATCCCATT

AAACTACAAACTTTCATTGCAAAGAGTATTGAGGATTTGCGAAAGAAAAACAGTGAATCA

TAA

>CjapCSP12

ATGGCTCGTCTAATCTGCACGATCGCGATCATTGGTATTGCACTGATGTGTGTACTTGCG

GAGGAAGAGAAATACGAGGATAAGTACGACGATATTGATGTCCACGAAGTACTTGAGAAC

GTTAAGCTACGAGAACAATATTATAAATGTTTCATGGCAACAGGACCATGCGTAACAGCA

GATCAGAAATTCTTCAGTAAGATCGTCAGTGAGGCTTTTCAAACTAAGTGCAAACTATGT

ACTGAAAAGCAAAAGTATATGTTGGACGAAATAAGTGAGTGGTATACGAAAAATGATCCT

GAAAAATGGAACGCATTTATTGCAAAGACTTTAGAAGATATGAAGAAAAAAGCTAAAGAA

TAA

>CjapCSP13

ATGGCTCGGTTAAATTGCATCATAATACTTATTAGTATTGCGTCGTGTGTTCTCGCAGAA

GAACTTTATAGCGATCAGTACGATCATATCGATGTGAATAATATTCTCAATAACGACAAA

CTACGGGACCAATATTTTAATTGTTATATGGAAACTGAACCATGTCTAACAGCAGAAGCA

AAATTTTATAGAGATATTGCTAGCGAAGCTTTACAAACAAAATGCAAAAGATGTACTGAA

AAGCAAAAGGAAATAATAGATGCAGTAGTTGATTGGTATACACAAAATAAACCTGACAAA

TGGCAGAAGATCGTTGAAAAAAGTTTAGAAGATATGAAAAAGAAGAATGCTGGTCAATAA
