# Supplementary material for: Antennal RNA-sequencing analysis reveals evolutionary aspects of chemosensory proteins in the carpenter ant, Camponotus japonicus
Source: Sci Rep. 2015 Aug 27;5:13541. doi: 10.1038/srep13541 (PMC4550911; doi:10.1038/srep13541)
Supplement: Supplementary DataSet 2 [file srep13541-s3.doc]

>CjapCSP1

MDKSSLCLLALGVLAAVIAEEMYSDMFDHINPDDILPNDELRNQYYNCFMDTGPCVTEDQ

KYFKEHAAEAFATKCRKCTEVQKKNVEKIVVWYTENRPQEWQAMVQKLMDDAKKLNIPFT

R*

>CjapCSP2

MALTIKFLILVCALFTATMAAESDNSEGQQSGRSRVSDEQLNIALSDKRYLTRQLKCALG

EAPCDPVGRRLKSLVPLVLRGSCPQCSPEETRQIKKVLSHIQRSFPKEWSRIVQQYAGVS

*

>CjapCSP3

MKFALVCLFAISTIVCVYGRPQDHYTDKFDNIDVDQILNNDRLLKRYVDCLLERSHVKCP

SEALELKKVLADAMATDCAKCTDRQKEIARKALDFLIINKTDMWNDLKSKYDPEEKYAKK

YEDRALKKEN*

>CjapCSP4

MKHLVVALITALSFSVVLAEDVQYTTKYDNIDVDAVINSERLLNGYVGCLLDRTPCTPDA

AELKKNLPDALEHDCAGCSEMQKNAADKISHHLIDNKPDDWRLLEDKYDPTGAYRRRYLE

NKSHEGGRLD*

>CjapCSP5

MNKQIIILIIIGSGLAVFCQAQDISSYLTDKRFIDKELHCLLETGDCDGFGKQIKRVLPV

VLKDKCRRCTPQQKANLHKLIQFLQSRYPTQWHTIEEMYSSPTFQ*

>CjapCSP6

MKKYLLISLASLMILVVATEKYTGKYDDVDVDKILQNNRVLNNYIRCLLDEGPCTAEGRE

LRKTLPDALSSSCSKCNDKQKATAEKVINHLKTKRSKDWDRLIAKYDPRGEYKKRYEQL*

>CjapCSP7

MKVLALLLIAVACALADDKYTTKFDNIDVDAILKSDRLLKNYVNCLLDKGNCTPDGKELK

EHLPDALETECSKCSEKQRTGTEKVIRFLVNKKPETWEQLKKKYDPNGEYSRRYEDEAEK

RNIKA*

>CjapCSP8

MKLPFLLLLSSFVFCGLVSGTENYTDIHDNVDIDAILNSDRLLKQYMDCILEKGSCTADA

RSLKRILPEAVATICEKCNLKQRQGARKIGNHLKKYKPELWTIFLEKYDPNKEYIENFEQ

FLAQVEE*

>CjapCSP9

MTKLVSCTFACLVMTLAVLIAHAEDEKYSSKYDHIDINEVLANSRLRNQYVRCLINISPC

TTGSARFLKDIQGEAFVTKCKKCTDKQIYILNAITDWFTKNEPETWNRMVQVAVEEAKRK

NA*

>CjapCSP10

MARPSYIVAIVVIALTCVLAEELYSSRFDDVDVRAIFNNAKLRNQYYNCFMDLSPCKTAD

QRFFKGIFSEALQSGCKRCTEKQKENLEIVLDWYTINDPIKLQTFIAKSIEDLRKKNSES

*

>CjapCSP12

MARLICTIAIIGIALMCVLAEEEKYEDKYDDIDVHEVLENVKLREQYYKCFMATGPCVTA

DQKFFSKIVSEAFQTKCKLCTEKQKYMLDEISEWYTKNDPEKWNAFIAKTLEDMKKKAKE

*

>CjapCSP13

MARLNCIIILISIASCVLAEELYSDQYDHIDVNNILNNDKLRDQYFNCYMETEPCLTAEA

KFYRDIASEALQTKCKRCTEKQKEIIDAVVDWYTQNKPDKWQKIVEKSLEDMKKKNAGQ*
